# Distraction osteogenesis for tibial nonunion with bone loss using combined Ilizarov and Taylor spatial frames versus a conventional circular frame

**DOI:** 10.1007/s11751-016-0264-4

**Published:** 2016-09-22

**Authors:** Ibrahim Elsayed Abdellatif Abuomira, Francesco Sala, Yasser Elbatrawy, Giovanni Lovisetti, Salvatore Alati, Dario Capitani

**Affiliations:** 1 Department of Orthopedic Surgery and Traumatology, Al-Azhar University Hospital, Assiut, Egypt; 2 Department of Orthopedic Surgery and Traumatology, Niguarda Hospital, Piazza Ospedale Maggiore 3, 20162 Milan, Italy; 3 Department of Orthopedic Surgery and Traumatology, Menaggio Hospital, Menaggio, CO Italy; 4 2 Nile Street, Sohâg, Egypt

**Keywords:** Bone transport, Tibial nonunion, Bone defect, Docking site, Taylor spatial frame, Ilizarov

## Abstract

This retrospective review assesses 55 tibial nonunions with bone loss to compare union achieved with combined Ilizarov and Taylor spatial frames (I–TSF) versus a conventional circular frame with the standard Ilizarov procedure. Seventeen (31 %) of the 55 nonunions were infected. Thirty patients treated with I–TSF were compared with 25 patients treated with a conventional circular frame. In the I–TSF group, an average of 7.6 cm of bone was resected and the lengthening index (treatment time in months divided by lengthening amount in centimeters) was 1.97. In the conventional circular frame group, a mean of 6.5 cm was resected and the lengthening index was 2.1. Consolidation at the docking site and at the regenerate bone occurred in 49 (89 %) of 55 cases after the first procedure. No statistically significant difference was shown between the two groups. Superiority of one modality of treatment over the other cannot be concluded from our data. Application of combined Ilizarov and Taylor spatial frames for bone transport is useful for treatment of tibial nonunion with bone loss.

*Level of evidence* Case series, Level III.

## Introduction

Treatment of segmental bone defects in the leg, especially those that are associated with soft tissue defects or an infection at the site of a nonunion, is challenging [1–4]. Treatment objectives include improvement in the quality of bone and soft tissue, correction of angulation and length, early mobilization to prevent stiff adjacent joints, promotion of union, and eradication of infection. The Ilizarov technique has improved limb reconstruction [5–8]. For small bone defects, the defect is compressed and osteotomy and lengthening are performed at the opposite end of the bone. With larger defects, lengthening and compression occur simultaneously such that the middle segment of bone is transported to fill the defect [2, 9, 10]. Once the defect has been closed, lengthening can be continued as required. The Ilizarov fixator also has been used to gradually close traumatic soft tissue defects [11]. Reconstruction is associated with longer rehabilitation time. Complications associated with bone transport and those occurring at the docking site might require additional surgical procedures and rehospitalization.

The Taylor spatial frame (TSF; Smith + Nephew, Inc., Memphis, TN USA) uses special struts and a computer program to calculate the position of imaginary “hinges” for simultaneous deformity correction in multiple planes and represents an advance in medicine and surgery. Although the TSF is more cumbersome than the standard Ilizarov frame (especially in diameter), it offers many advantages, including reliability and the ability to simultaneously correct rotation, angulation, and translation deformities (six-axis deformity correction) without the need to apply rotational devices or to change hinge placement, as usually is necessary with the standard Ilizarov frame [12]. Primary fixation and definitive fixation with the TSF are effective. Advantages include continuity of device until union, reduced risk of infection, early mobilization, restoration of primary defect caused by bone loss, easy and accurate application, convertibility and versatility compared with a monolateral fixator, and improved union rate and range of motion for lower extremity long-bone fractures in patients with multiple traumatic injuries [13].

Our study presents outcomes of the combined Ilizarov frame and TSF (I–TSF) compared with a standard Ilizarov procedure and a conventional circular frame for correcting segmental tibial defects [9, 10]. The current study was approved by the ethical committee at our hospital.

## Patients and methods

We performed a retrospective, case-matched comparison of patients who underwent tibial deformity correction with I–TSF and those who underwent correction with a conventional circular frame during tibial bone transport. Allocation of type of frame was based on medical necessity, with simpler cases of nonunion with bone loss receiving conventional circular frames and more complex cases that included rotation, angulation, and/or translation deformity receiving I–TSF. Our study group was a retrospective cohort of 55 patients with tibial nonunions and bone loss treated with bifocal and trifocal techniques during the period from 1999 through 2011. The demographics and clinical features of the 55 patients are presented in Table 1.

**Table 1:** Study population demographics

	Overall population (*n* = 55)	Group A (*n* = 30)	Group B (*n* = 25)
Age, year, mean ± SD (range)	41.5 ± 18 (15–79)	39 ± 20.4 (15–79)	44.5 ± 14.6 (21–75)
Sex, *n* (%)			
Male	44 (80)	25 (83)	19 (76)
Female	11 (20)	5 (17)	6 (24)
Local infection, *n* (%)	38 (69)	20 (67)	18 (72)
Bone transport, cm, mean ± SD (range)	7.1 ± 3.3 (3–17)	7.6 ± 3.5 (3–15)	6.5 ± 3 (3–17)
Treatment type, *n* (%)			
Trifocal	29 (53)	20 (67)	9 (36)
Bifocal	26 (47)	10 (33)	16 (64)
External fixation time, d, mean ± SD (range)	391 ± 140.5 (120–770)	418 ± 144.8 (168–770)	359 ± 130.8 (120–670)
Lengthening index, mo/cm, mean ± SD (range)	2 ± 0.8 (1.1–4)	1.97 ± 0.7 (1.1–3.4)	2.1 ± 0.9 (1.3–4)
Mean union rate after first surgery	89	90	88
Duration of follow-up, days, mean ± SD (range)	50 ± 14.7 (25–78)	48 ± 12.8 (26–78)	53 ± 16.5 (25–74)

None of the differences shown reached statistical significance

*SD* standard deviation

**Fig. 1:**
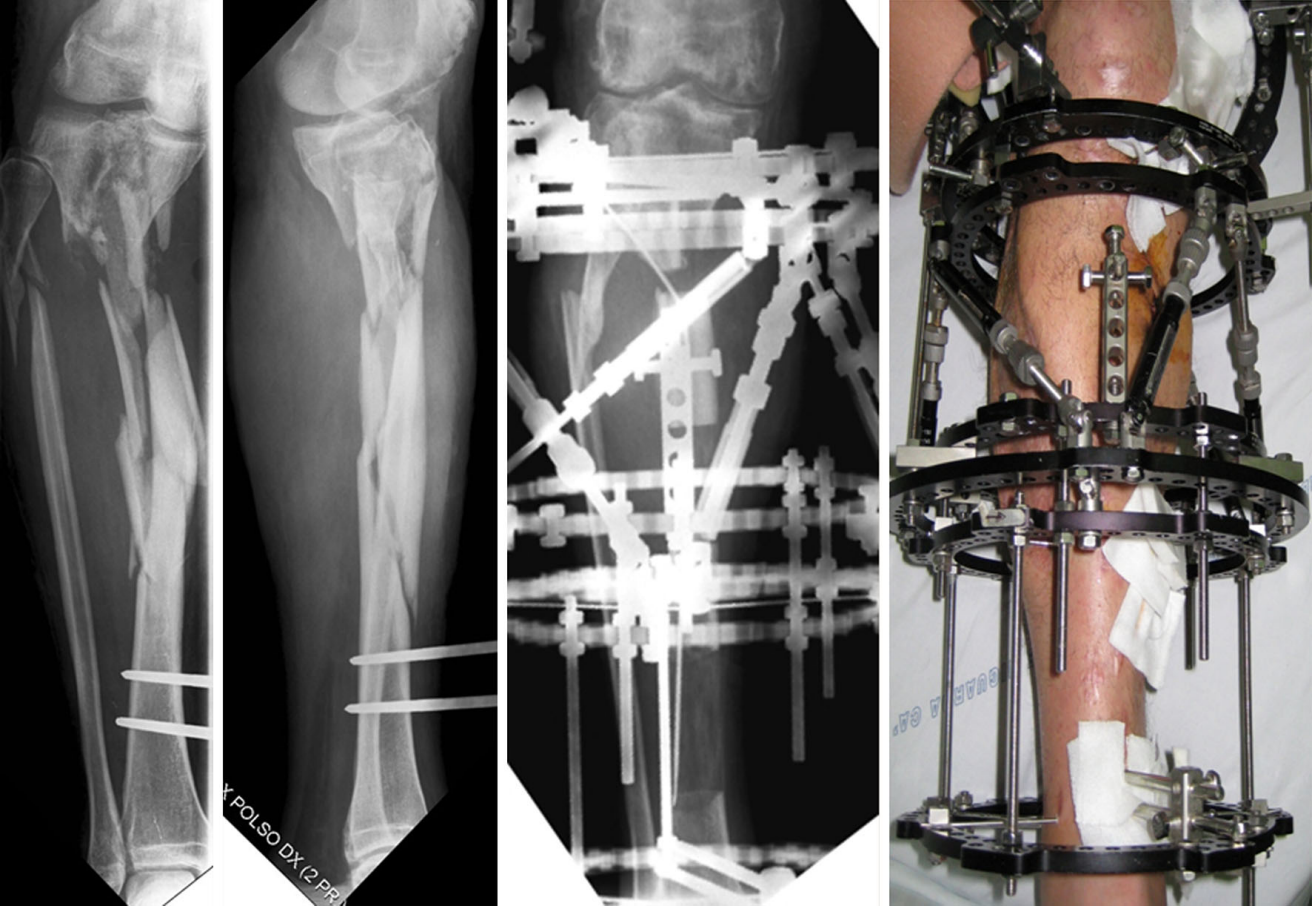
43-year-old man with infected tibial nonunion treated with bony resection of all infected bone and a trifocal retrograde tibial bone transport. From *left to right*, images show radiographs of the tibial nonunion with a temporary external fixator, anteroposterior radiograph with the TSF applied, and clinical photograph after application of the TSF during tibial bone transport

Combined I–TSF was applied to 30 patients (25 male and five female patients), with a mean age of 39 years (age range 15–79 years) (group A). Local infection was present in 20 (67 %) of 30 cases. Bifocal transport was used in 10 (33 %) of the group A patients and trifocal in 20 (67 %) (Fig. 1). Refreshing procedure at the docking site with autologous bone grafting was performed in 24 (80 %) cases. Fibular osteotomy was performed in 20 (67 %) of 30 patients. Tendo-Achilles lengthening was performed in six (20 %) patients.

A conventional circular frame was used for 25 patients (19 men and six women) with a mean age of 44.5 years (age range 21–75 years) (group B). The standard Ilizarov frame (Sintea Plustek, Assago, Italy) was used in 10 patients, the TrueLok frame (Orthofix, McKinney, TX USA) in eight, the Sheffield frame (Orthofix) in five, and the full ring fixator (Synthes Gmbh, Solothurn, Switzerland) in two. The standard Ilizarov procedure was used with all four types of conventional circular frames. Local infection was present in 18 (72 %) of 25 cases. Bifocal transport was performed in 16 (64 %) patients and trifocal transport in nine (36 %). Refreshing procedure at the docking site with autologous bone grafting was performed in nine (36 %) cases. Fibular osteotomy was performed in 14 (56 %) of 25 patients.

All patients were encouraged to bear partial weight progressively with crutches on the second day after surgery. Quadriceps isometric exercises were started immediately after the operation to maintain or increase muscle strength. Range-of-motion exercises of the knee were initiated as soon as the comfort of the patient allowed. The TSF was removed when at least tricortical consolidation was seen on anteroposterior and lateral view radiographs before complete removal of the frame. The fixators were slowly destabilized by removing struts or bars over a period of 3 weeks. After frame removal, patients were restricted to partial weight bearing for 4–6 weeks and no brace was used. Full weight bearing was allowed between the 4th and 10th postoperative week, based on clinical and radiological evidence of healing at the nonunion site and at the site of lengthening and deformity correction.

Patients’ data were collected from medical records and radiographs that were obtained every 2 weeks during the distraction phase and once a month during the consolidation phase. Complications encountered intraoperatively and during treatment were also recorded. With use of the classification system presented by Paley [14], minor complications were problems that did not require additional surgery, major complications were obstacles that resolved with additional surgery, and true complications were sequelae that remained unresolved at the end of the treatment period. Preoperative and last follow-up radiographic measurements were reviewed for all patients. External fixation time (length of time with the frame applied), lengthening index (treatment time in months divided by lengthening amount in centimeters), amount of obtained length, and segment transfer were all calculated.

### Operative technique

All nonunions were treated with radical bony resection of all necrotic bones and bone transport according to Ilizarov distraction osteogenesis principles. The TSF rings were placed on the proximal and distal fragments parallel to their respective joints, allowing adequate soft tissue clearance. The frame was mounted orthogonally to the mechanical axis of the tibia and fixed initially with two wires, one proximal and one distal. Additional wires and half-pins were then inserted, aiming for at least three points of fixation both proximally and distally. Great care must be taken to keep the master tab area of each TSF ring free for future strut applications. Six-millimeter hydroxyapatite (HA)-coated half-pins (Orthofix) were used in all patients [15].

For proximal and distal tibial nonunions, the constructs were extended to the distal femur or to the foot to increase frame stability. The total residual computer program of the TSF was used to restore the normal limb axis and to achieve lengthening if necessary. A percutaneous Gigli saw osteotomy of the tibia was made through two transverse incisions of the skin in both groups. The latency period before starting distraction osteogenesis was 12–14 days. Distraction ranged from 0.5 to 1.5 mm/day, depending on the regenerative quality and the number of sites of osteotomy. When bone capitation at the docking site was achieved, inter-fragmentary compression was continued at the rate of 0.25 mm/day for 5–7 days. Once consolidation had commenced, the rate was 0.25 mm every 2 weeks for more 2 months. Standard pin care with possible showering and application of dry gauze around the pins was recommended [13, 16]. Oral antibiotics were prescribed for patients with pin site infections.

All patients were encouraged to partially bear weight with the assistance of crutches on the 2nd day after surgery. All frames were dynamized before removal. Group A dynamization was performed by replacing the TSF struts with traditional Ilizarov rods. The HA-coated half-pins were removed with the patient under short-term sedation in the operating room. After frame removal, patients were restricted to partial weight bearing for 4–6 weeks. The final bony and functional results were classified accordingly to the criteria proposed by Paley and Maar [18].

### Statistical analysis

Obtained data are presented as means ± standard deviations, ranges, numbers, and percentages. Results were analyzed by conducting one-way analysis of variance with post hoc Tukey honest significant difference test and Chi-squared test. Statistical analysis was conducted by using SPSS version 15 statistical software package (IBM Corporation, Armonk, NY). A *p* value < 0.05 was considered statistically significant.

## Results

The mean duration of follow-up was 48 ± 12.8 months (range 26–78 months) in group A and 53 ± 16.5 months (range 25–74 months) in group B, with a nonsignificant difference in favor of group B (*p* > 0.05). Positive nonsignificant correlation was shown between presence of infection and length of duration of follow-up in both groups (*p* > 0.05). Table 2 presents the postoperative bony and functional outcomes of the study population.

**Table 2:** Postoperative bony and functional outcomes

Outcomes	Overall population (*n* = 55)	Group A (*n* = 30)	Group B (*n* = 25)
Bony, *n* (%)			
Excellent	28 (51)	17 (47)	11 (44)
Good	18 (33)	10 (33)	8 (32)
Fair	5 (9)	2 (7)	3 (12)
Poor	4 (7)	1 (3)	3 (12)
Functional, *n* (%)			
Excellent	25 (45)	14 (47)	11 (44)
Good	21 (38)	12 (40)	9 (36)
Fair	5 (9)	3 (10)	2 (8)
Poor	4 (7)	1 (3)	3 (12)

None of the differences shown reached statistical significance

**Fig. 2:**
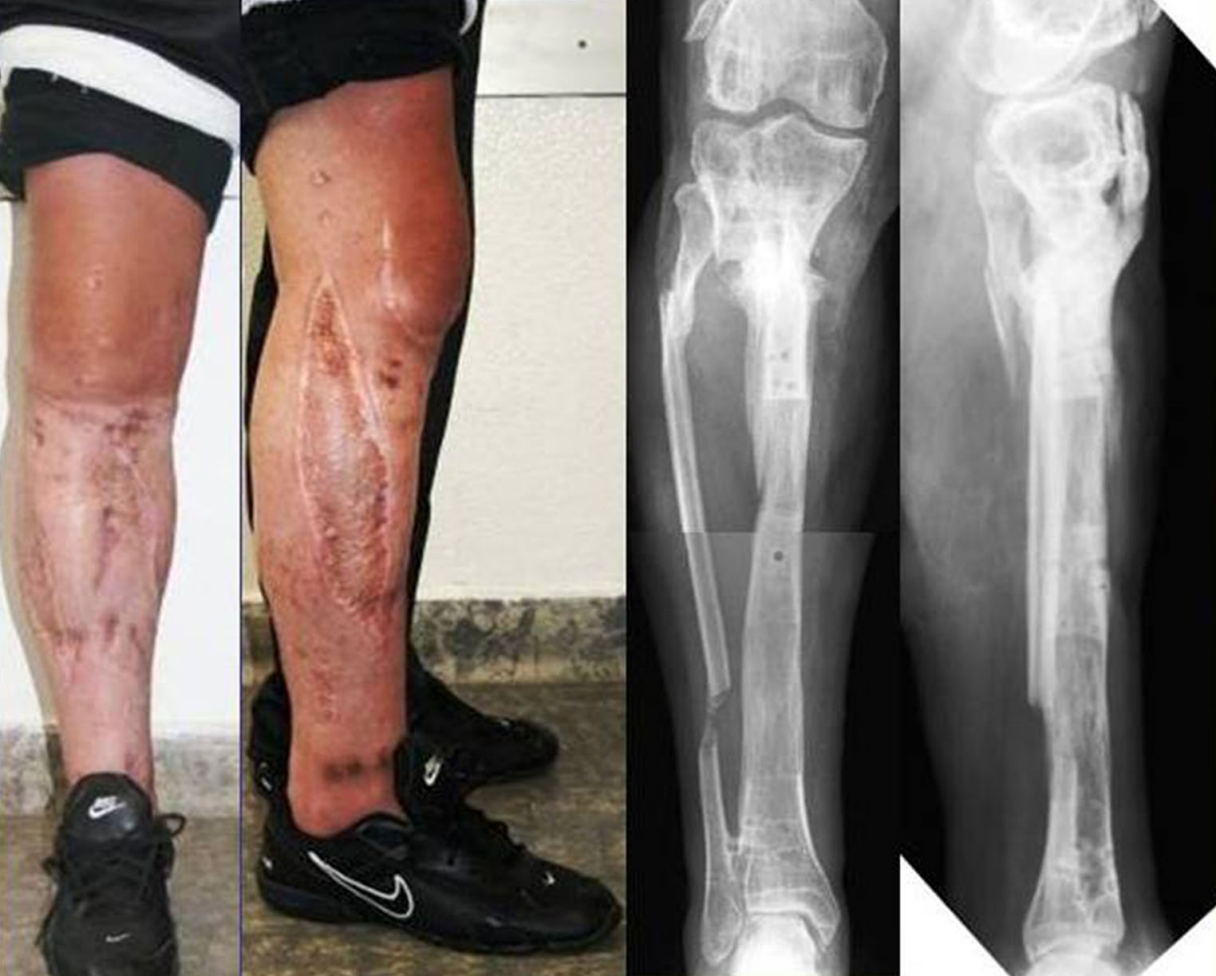
Clinical and radiographic follow-up images obtained 3 years after tibial frame removal. Treatment time, 16 months; lengthening amount, 140 mm; lengthening index (months/cm), 1.14. Mechanical axis deviation was 8 mm medial to the center of the knee joint line. Patient resumed full weight bearing without support and with no discomfort

In group A, tibial bone healing was achieved in all cases (100 %), with a union rate of 90 % after the first procedure. The mean external fixation time was 418 ± 144.8 days (range 168–770 days). The average distance of bone transport was 7.6 ± 3.5 cm (range 3–15 cm). The mean lengthening index was 1.97 ± 0.7 (range 1.1–3.4) (Fig. 2). At the time of the 3-year follow-up visit, the fracture sites were completely united and the patients had no clinical infection, skin defect, or limb length discrepancy. Using the Association for the Study and Application of the Method of Ilizarov outcome score, the bony result was excellent and the functional result was good. Bony results were excellent in 17 cases, good in 10, fair in two, and poor in one. Functional results were excellent in 14 cases, good in 12, fair in three, and poor in one. Negative nonsignificant correlation was shown between lengthening index and both external fixation time and distance of bone transport.

In group B, tibial bone healing was achieved in 24 (96 %) of 25 cases, with a union rate of 88 % after the first surgery with a nonsignificant difference in favor of group A (*p* > 0.05). The mean external fixation time was 359 ± 130.8 days (range 120–670 days), which was nonsignificantly shorter than the external fixation time in group A (*p* > 0.05). The average distance of bone transport was 6.5 ± 3 cm (range 3–17 cm), which was shorter than the average distance reported in group A, but the difference did not reach statistical significance (*p* > 0.05). The mean lengthening index was 2.1 ± 0.9 (range 1.3–4.0) and was nonsignificantly higher than the index reported for group A (*p* > 0.05). Bony results were excellent in 11 patients, good in eight, fair in three, and poor in three. Functional results were excellent in 11 patients, good in nine, fair in two, and poor in three. Bony and functional results were nonsignificantly lower in group B compared with group A (*p* > 0.05). A negative nonsignificant correlation was shown between lengthening index and both external fixation time and distance of bone transport (*p* > 0.05).

In both groups, negative nonsignificant correlation was shown between lengthening index and external fixation time (*p* > 0.05). Likewise, in both groups, negative nonsignificant correlation was shown between lengthening index and distance of bone transport (*p* > 0.05).

### Complications

No intraoperative complications were caused by insertion of the pins or use of the Gigli saw. No compartment syndrome occurred in association with tibial osteotomy. In both groups, pain was the most common complaint during the distraction period, particularly in patients requiring lengthening in excess of 4 cm. Pain was relieved by orally administered analgesics. The most frequently occurring complication in our study was pin tract infection, which occurred in 31 patients in both groups (56 %).

Other minor complications occurred in group A: 1) half-pin breakage occurred in three patients and half-pin loosening, requiring early removal, in two; 2) residual limb length discrepancies measuring 1.5 cm occurred in two patients and 2.0 cm in one (treated with internal shoe lifts, no functional problems); 3) minimal (<5°) regenerate bending occurred in three patients.

Seven major complications occurred in group A: 1) osteitis occurred in the distal tibia of one patient 3 months after fixator removal (healed with arthrodesis of the ankle after two repeated bifocal bone transports); 2) bending of regenerate bone occurred in two patients (both recovered after additional surgical procedures: reapplication of fixator for 3 months in one and plate fixation in the other; 3) uncommon delayed peroneal artery pseudoaneurysm occurred in one patient after surgical procedure at the docking site (supported by angiography, embolization with coil treatment was successful) [17]; 4) equinus ankle contractures occurred in three patients with large bone defects (trifocal bone transports: two retrograde and one antegrade). Correction was obtained with Achilles tendon lengthening and was maintained with extension of the frame to the foot.

Minor complications occurred in group B: 1) three pins fractured in three patients; 2) five pins were added during the course of treatment of three patients to provide additional function; 3) minimal (<5°) regenerate bending occurred in four patients; 4) limb length discrepancies measuring 1.5 and 2.0 cm occurred in two patients without causing functional problems.

Four major complications occurred in group B: 1) refracture of previously consolidated docking sites occurred in two patients at 318 and 121 days because of recurrent sepsis (both treated with second bifocal treatment with simple compression at docking site: one healed with optimal bony and functional results, the other, a 61-year-old man who was diabetic and a heavy smoker with an initial septic nonunion of the leg, was still receiving treatment at the time of this writing; 2) nonunion of the regenerate bone in an immunosuppressed patient who was a heavy smoker and who otherwise achieved consolidation of the docking site (further treatment was refused); 3) equinus ankle contracture occurred in one patient (correction obtained with Achilles tendon lengthening and extension of the frame to the foot); 4) misalignment of the transported distal fragment before docking in one patient (required additional correction surgery).

## Discussion

The bifocal and trifocal bone transport using the I–TSF technique in group A produced excellent and good bony and functional results, respectively, in 27 and 26 cases, respectively, versus 19 cases in group B. In three (10 %) cases in group A, previous treatment had failed compared with three (12 %) cases in group B. The treatment times with the bifocal and trifocal techniques were long in both groups. Considering the intrinsically long treatment times, careful patient selection is necessary.

In a recent review of our experience [10], we assessed and compared I–TSF trifocal and bifocal techniques for the treatment of seven segmental tibial bone defects, achieving union without malalignment of the mechanical axis [19]. With this report, we updated our series with 18 new cases, introducing a second group of 25 cases in which a bone transport procedure was performed with a conventional circular frame. These results represent the early experience with use of the TSF with this technique. As time has progressed, the technique has been refined and results have become more reliable. In the present study, no case developed malalignment or bony deformity in either group.

Bone transport is inherently more complicated than compression–distraction, with respectively longer treatment times and further operative procedures being necessary. Because the defect is closed gradually, a time delay exists before bony contact and compression occur at the docking site, thus prolonging treatment time. As noted by Paley and Maar [18], the bone healing index gradually decreases the longer the lengthening time and/or the larger bone transport gap is. The transported segment of bone can be deviated as it passes through the soft tissues, leading to translation at the docking site.

In the three-dimensional space, six different directions of displacement are possible between an upper and a lower ring: the six degrees of freedom. The TSF allowed the necessary ring displacements in all cases without time-consuming preoperative planning of joint or slider positions using the software mode of the total residual program [20].

Treatment of rotation deformities with respect to the vertical axis is known to be especially difficult. With the TSF, rotation with respect to any axis in space can be performed, and translations attributable rotations can be taken into account mathematically [21]. In bifocal and trifocal transports, strut bars of TSF can interfere with half-pins during bone transfer or axial and rotational corrections. Strut bars allow precise docking of the bone transport to the target point, with accurate axis alignment and, when resections are correctly performed, circumferential compression of the docking site [18]. At times, partial remounting of the fixator is required during the course of treatment.

We did not treat bone loss with acute shortening and re-lengthening for immediate contact of the resected ends because infection was present in 38 (69 %) of 55 cases and the bone defects were larger than 3 cm in all patients. Bone grafting at the docking site was required in 33 (60 %) of 55 cases of bone transport [22, 23]. Consolidation of the regenerate bone without further complications was achieved in 28 (93 %) of 30 patients in group A and 24 (96 %) of 25 patients in group B. Consolidation of the docking site without further complications was achieved in 29 (97 %) of 30 patients in group A and 23 (92 %) of 25 patients in group B. Percentages of healing were therefore similar. Group B patients, however, had shorter transports (6.5 versus 7.6), and this factor could be a bias affecting the results of group A, as has been observed in terms of total external fixation time in different groups. In addition, the lengthening index seems to be superior in group B (2.10 versus 1.97 in group A), but the difference is largely because of a higher number of trifocal procedures. Several complications occurred in our study; however, the rate was reasonable considering the complexity of the cases.

One limitation of our study was the variety of fixators used in group B. Four types of conventional circular frames were included. However, the standard Ilizarov procedure was used with all four types. Also, cases that were allocated to receive I–TSF were more complex cases than those receiving only conventional circular frames, which might have introduced selection bias. Further limitations of our study include the small sample size and retrospective design. Further comparative studies are needed to prove the efficacy of bone transport with a TSF in combination with an Ilizarov frame compared with a conventional circular frame only.

## Conclusion

When it is necessary to perform bone transport, to optimize conditions for healing, the necrotic or infected bone ends should be resected and fashioned in such a way as to enhance docking. The frame should be carefully mounted to be parallel in both planes to prevent translation. Bone grafting of the docking site, if necessary, should be performed early. Our results are promising in terms of achieved union rates, axis alignment of the lower extremity, and eradication of infections. Although the superiority of one treatment modality over the other cannot be concluded based on our data, the study shows that the combined use of the TSF and Ilizarov frame for bone transport is useful for treatment of tibial nonunion with bone loss.
